# Adult Pancreatic Hemangioma: Case Report and Literature Review

**DOI:** 10.1155/2009/839730

**Published:** 2009-05-03

**Authors:** Gerhard S. Mundinger, Shannon Gust, Shien T. Micchelli, Elliot K. Fishman, Ralph H. Hruban, Christopher L. Wolfgang

**Affiliations:** ^1^Division of Plastic, Reconstructive, and Maxillofacial Surgery, The Johns Hopkins Hospital, Baltimore, MD 21218, USA; ^2^Johns Hopkins Medical School, The Johns Hopkins Hospital, Baltimore, MD 21218, USA; ^3^Department of Pathology, The Johns Hopkins Hospital, Baltimore, MD 21218, USA; ^4^Department of Radiology, The Johns Hopkins Hospital, Baltimore, MD 21218, USA; ^5^The Sol Goldman Pancreatic Cancer Research Center, The Johns Hopkins Hospital, Baltimore, MD 21218, USA; ^6^Department of Surgery, The Johns Hopkins Hospital, Baltimore, MD 21218, USA

## Abstract

We report an adult pancreatic hemangioma diagnosed on pathological specimen review following pylorus preserving pancreaticoduodenectomy for a symptomatic cystic mass in the head of the pancreas. Eight cases of adult pancreatic hemangioma have been reported in literature since 1939. Presenting symptoms, radiographic diagnosis, pathologic characteristics, and treatment of adult pancreatic hemagiomas are discussed following review of all published cases.

## 1. Introduction

Hemangiomas, while common in the liver, are rarely found in the pancreas. Few cases of pancreatic hemangioma presenting in adulthood have been documented. Hemangiomas are rarely suspected clinically due to their nonspecific symptoms. As a result, most are diagnosed incidentally following resection or attempted resection for symptomatic cystic pancreatic masses identified on ultrasound (US), angiography, CT, or magnetic resonance imaging (MRI). We report an adult patient with a pancreatic hemangioma diagnosed histologically following pylorus preserving pancreaticoduodenectomy (PPPD) for a symptomatic cystic lesion in the head of the pancreas. 

## 2. Case Report

**Figure 1:**
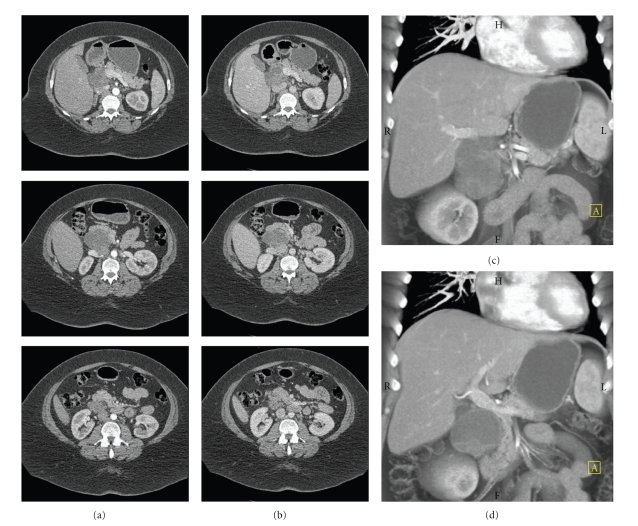
Abdominal CT scan with IV contrast demonstrating a cystic mass in the head of the pancreas. (a) Arterial phase, (b) venous phase, (c) three dimensional coronal CT reconstruction of the celiac axis illustrating resectability, and (d) three dimensional CT reconstruction of the porta hepatis.

A 45-year-old morbidly obese woman with no significant past medical history presented to her primary care physician with 3 months of stabbing epigastric pain radiating through to her back. She was found to have a large pancreatic mass on ultrasound. A computed tomography (CT) scan at our institution demonstrated a 6.2 by 5.3 cm mass in the pancreatic head (Figures 1(a) and 1(b)). On arterial phase imaging, the mass was of low density relative to the pancreas without marked contrast enhancement. No adenopathy was apparent. The mass was distinct from the mesenteric vessels and therefore likely resectable (Figures 1(c) and 1(d)) [1, 2]. Her serum CA 19-9 was 7.2 U/mL. Her case was reviewed at our multidisciplinary pancreatic cancer conference (http://pathology.jhu.edu/pancreas/MDC/index.html) and was felt to be a benign lesion. The differential diagnosis included duplication cyst, paraganglioma, or cystic gastrointestinal stromal tumor. 

Given the patient's symptoms, she was offered surgical resection. Intraoperatively, the mass was found to be inseparable from both the duodenum and the head of the pancreas. Therefore the patient underwent a pylorus preserving pancreaticoduodenectomy without incident. Intraoperative frozen-section revealed a benign cystic lesion. The patient's postoperative course was uneventful, and she was discharged to home on postoperative day nine. 

**Figure 2:**
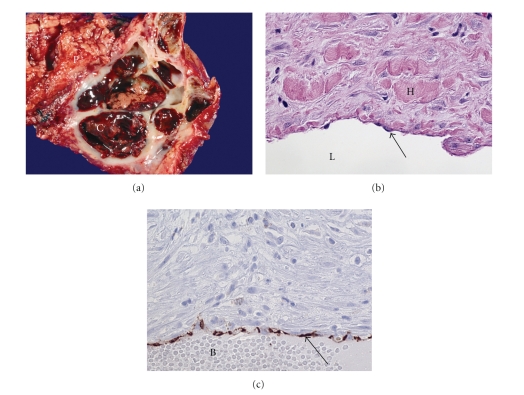
(a) Gross pathology specimen. 5.5 cm hemangioma containing denuded multiloculated cysts (C) with intracystic hemorrhage. Pancreatic acinar tissue (P). (b) Hematoxylin and eosin stain (160x) of specimen demonstrating cyst lumen (L) lined with endothelial intima (arrow) and surrounded by media rich in hyalinized collagen (H). (c) CD31 stain (160x) of specimen with strong staining of endothelial intima (arrow). Blood in cyst lumen (B).

Gross pathologic examination revealed a 5.5 cm hemangioma predominantly composed of denuded multiloculated cysts containing intracystic hemorrhage (Figure 2(a)). On microscopic examination, the cysts were lined by a single layer of uniform flattened cells. Immunolabeling revealed that this lining was positive for CD 31, focally positive for CD34 and negative for cytokeratin (AE1/AE3), supporting the diagnosis of hemangioma (Figures 2(b), 2(c)). The lesion had “pushing” rather than infiltrative borders, and the endothelial cells displayed uniform nuclei without atypia. The stroma was composed of dense hyalinized collagen. All nodes and margins were negative for tumor. Background pancreatic tissue demonstrated pancreatic intraepithelial neoplasm-PanIN-1B [3]. 

## 3. Discussion

Pancreatic vascular neoplasms, including lymphangioma, hemangioma, hemolymphangioma, hemangiopericytoma, hemangioblastoma, and hemangiosarcoma are cystic lesions of the pancreas, collectively accounting for 0.1% of all pancreatic tumors [4]. Pancreatic hemangiomas are an extremely uncommon benign pancreatic vascular neoplasm. Adult pancreatic hemangiomas are a different pathologic entity from those that arise in the pediatric age group [5]. Pediatric (infantile) pancreatic hemangiomas undergo proliferation in infancy, only to slowly involute and regress over several years, leaving a fibro-fatty residuum by adulthood [6]. 

Only nine cases of adult pancreatic hemangiomas have been reported in literature since 1939 (Table 1) [7–14]. Although 5 potential cases were reported before 1939, these reports were unavailable for review [15–19]. In our institutional pancreatic database, containing over 3000 resected pancreatic specimens, we found no additional hemangiomas.

As in our case, most patients with pancreatic hemangioma present with vague abdominal pain, although one case presented with melena and hematemesis, and another with nausea and thrombocytopenia [8, 10]. Diagnostic imaging modalities used to aid in diagnosis illustrate the expected use of contemporarily available state-of-the-art imaging modalities, from plain films in the 1960s to MRI and three-dimensional multiplanar CT reconstruction today. 

Typically, hemangiomas are strongly contrast enhancing in the arterial phase of conventional contrast-enhanced CT imaging [11]. However, cystic tumors of the pancreas often contain areas of neovascularization with arteriovenous shunting, and blood flow through these cavernous vascular components is slow [11, 13]. This can result in diminished contrast enhancement on arterial phase CT. The ratio of cystic to solid tissue in the neoplasm also influences the relative degree of tumor vascularity, which could also influence expected arterial phase signal intensity [20]. As our case did not demonstrate the contrast-enhanced CT features typical of a hemangioma, this was not included in our initial differential. We agree with Kobyashi et al. and Chang et al. that poor arterial phase enhancement on three-phase contrast-enhanced CT cannot rule out pancreatic hemangioma [11, 13]. This is supported by the fact that three of four pancreatic hemangiomas imaged with contrast-enhanced CT did not show marked arterial phase enhancement [11, 13, 14]. Low T1w signal attenuation with high T2w signal attenuation on unenhanced MRI has been purported as a corroborative finding for pancreatic hemangioma [11].

Pathologically, the specimen was typical of a cavernous hemangioma with blood filled spaces separated by fibrous connective tissue [21]. Immunohistochemistry showed that the neoplastic cells expressed the endothelial markers CD31 and CD34, confirming that the neoplasm was of vascular endothelial origin. CD 31 and CD34 immunohistochemical labeling has been previously reported for a pediatric pancreatic hemangioma [5]. In the adult literature, labeling with antibodies to factor VIII-related antigen, a well-established marker for vascular endothelium, has been reported [13]. This is the first case of CD31 and CD34 labeling of an adult pancreatic hemangioma reported in literature. 

Treatment of pancreatic hemangiomas has been variable (Table 1). Generally, a conservative approach is justified for hemangiomas given the benign nature of the lesion. However, there is a risk of bleeding, and this has been a presenting sign [8]. In this case reported by Ringoir et al., definitive resection was avoided given the patient's age and the considerably higher morbidity and mortality of pancreatic resection at the time of operation in 1959 when compared to today [22].This is the first reported case of pylorus preserving pancreaticoduodenectomy for pancreatic hemangioma.

## 4. Conclusions

Hemangiomas are rare lesions of the pancreas and are often not suspected clinically. This case of an adult pancreatic hemangioma is the ninth reported in literature since 1939 and is the first treated with a pylorus preserving pancreaticoduodenectomy. Review of all reported cases and relevant literature reveals that, in contrast to other hemangiomas, pancreatic hemangiomas may not contrast enhance on arterial phase CT imaging. This modality is therefore an ineffective means for ruling out pancreatic hemangioma. Understanding of the pathophysiology and natural history of these lesions remains in its infancy. 

## Figures and Tables

**Table 1:** Adult pancreatic hemangiomas reported in literature since 1939.

Case	Year	Authors	Age	Sex	Presentation	Diagnostic imaging	Location/Size	Treatment	Pathologic Description	IHC* Positivity
#1	1939	Ranström [7]	61	F	Found incidentally at autopsy	—	Head 7 × 7 cm	—	Pancreatic hemangioma	—
#2	1961	Ringoir et al. [8]	71	F	Hemetemesis, melena	Abdominal plain film, intravenous cholangiography	Head 15 cm diameter	Retrocolic gastroenterostomy, vagotomy	Pancreatic hemangioma	—
#3	1972	Colardyn et al. [9]	42	M	Abdominal/back pain	Abdominal plain film, angiography	Body/tail	Fat free diet; anticholinergics	—	—
#4	1985	Mangin et al. [10]	62	F	Malaise, nausea, thrombocytopenia	US, ERCP, CT (non-contrast), arteriography	Head/body/tail 20 × 7 cm	Laparotomy, observation	Pancreatic hemangioma	—
#5	1991	Kobayashi et al. [11]	30	M	Abdominal distention	US, CT, angiography, MRI	Head 20 cm greatest dimension	Pancreatico-duodenectomy	Pancreatic hemangioma	—
#6	1991	Dageförde et al. [12]	79	F	Abdominal pain	US, ERCP, angiography	Body/tail junction 6 × 3 cm	Observation	—	—
#7	2003	Chang et al. [13]	70	F	Epigastric tenderness	CT, angiography	Body/tail junction 4 × 3.2 cm	Subtotal pancreatectomy	Pancreatic hemangioma	Factor VIII-related antigen
#8	2006	Plank et al. [14]	36	M	Abdominal pain	CT, MRI, intraoperative US	Head 3 cm greatest dimension	Laparotomy, observation	—	—
#9	2008	Mundinger et al.	45	F	Epigastric pain radiating through to back	CT, MRI	Head 6.2 × 5.3 cm	Pylorus preserving pancreatico-duodenectomy	Pancreatic hemangioma	CD 31CD 34

*Immunohistochemistry.
